# 4-Octyl-itaconate and dimethyl fumarate inhibit COX2 expression and prostaglandin production in macrophages

**DOI:** 10.4049/jimmunol.2100488

**Published:** 2021-10-11

**Authors:** Ciana Diskin, Alessia Zotta, Sarah E. Corcoran, Victoria J. Tyrrell, Zbigniew Zaslona, Valerie B. O’Donnell, Luke A.J. O’Neill

**Affiliations:** 1 School of Biochemistry and Immunology, Trinity Biomedical Sciences Institute, Trinity College, Dublin 2, Ireland; 2 Systems Immunity Research Institute, School of Medicine, Cardiff University, Heath Park, Cardiff, CF14 4XN, UK

## Abstract

Prostaglandins (PGs) are important proinflammatory lipid mediators, the significance of which is highlighted by the widespread and efficacious use of non-steroidal anti-inflammatory drugs (NSAIDs) in the treatment of inflammation. 4-Octyl itaconate (4-OI), a derivative of the Krebs cycle-derived metabolite itaconate, has recently garnered much interest as an anti-inflammatory agent. Here we show that 4-OI limits PG production in macrophages stimulated with the Toll-like receptor 1/2 (TLR1/2) ligand Pam3CSK4. This decrease in PG secretion is due to a robust suppression of COX2 expression by 4-OI, with both mRNA and protein levels decreased. Dimethyl fumarate (DMF), a fumarate derivative used in the treatment of multiple sclerosis (MS), with properties similar to itaconate, replicated the phenotype observed with 4-OI. We also demonstrate that the decrease in COX2 expression and inhibition of downstream prostaglandin production occurs in an NRF2-independent manner. Our findings provide a new insight into the potential of 4-OI as an anti-inflammatory agent and also identifies a novel anti-inflammatory function of DMF.

## Introduction

Prostaglandins are key lipid mediators, which exert a wide variety of physiological roles. Their synthesis begins with the release of arachidonic acid by cytoplasmic phospholipase A2 (cPLA2) from membrane phospholipids, after which it is converted to prostaglandin H_2_ (PGH_2_) by the cyclooxygenase enzymes. COX1 is constitutively and ubiquitously expressed and is known to play several homeostatic roles, while COX2 is inducible by inflammatory stimuli including Toll-like receptor (TLR) ligands and cytokines. PGH_2_ can then be converted into various prostaglandins (PGs) and thromboxanes by a range of synthase enzymes (1).

The widespread and effective use of NSAIDs, which inhibit COX enzymes, highlights the clinical importance of blocking prostaglandin production in inflammation (2). The proinflammatory effects of PGE_2_ in particular are well characterised. The capacity of PGE_2_ to induce a wide range of physiological and often pathological phenotypes is largely due to its binding to four different receptors (EP1-4), which vary in their tissue expression and downstream signal transduction pathways (3). PGE_2_ has been shown to activate mast cells (4), T helper 1 (Th1) cells (5, 6) and Th17 cells (7–9). PGE_2_ has been implicated in inflammatory diseases such as psoriasis (9) and rheumatoid arthritis (10), in pain responses (11, 12) and recently in aging (13). However PGE_2_ has also been shown to exert anti-inflammatory functions, particularly in the environment of the lung (14–16). Another prostaglandin, PGD_2_, has been reported to contribute to the allergic response. Engagement of PGD_2_ with its receptors facilitates chemotaxis and activation of eosinophils and Th2 cells during allergic disease (17–19). Thromboxanes, which are also downstream of COX activity, promote vasoconstriction and platelet aggregation (20).

Itaconate is a metabolite that has emerged in recent years as an important immunomodulator (21). It is synthesised via the decarboxylation of the Krebs cycle intermediate *cis*-aconitate by the enzyme aconitate decarboxylase 1 (ACOD1, also known as IRG1), encoded by *immune responsive gene 1 (Irg1)* (22). The expression of IRG1 is predominantly restricted to macrophages and several other immune cell types and is markedly upregulated upon stimulation with TLR ligands (23), thereby leading to an increase in intracellular itaconate. 4-Octyl itaconate (4-OI) is a cell-permeable derivate that is commonly used in the study of itaconate and has been shown to be converted into itaconate intracellularly in macrophages (24).

An important aspect of itaconate biology is that both the endogenous metabolite and 4-OI have been shown to function as cysteine modifiers (25). This cysteine alkylation was originally termed 2,3-dicarboxypropylation and is also referred to as itaconation. This post-translational modification (PTM) is the basis of many of the anti-inflammatory functions associated with itaconate and 4-OI (24, 26–30). 4-OI has been shown to modify cysteine residues on KEAP1 (25), which functions as a negative regulator of the master antioxidant transcription factor NRF2. Modifications of these cysteine residues cause KEAP1 to be degraded, which liberates NRF2 and permits translocation to the nucleus (31), where NRF2 induces transcription of antioxidant genes (32) and inhibits transcription of certain pro-inflammatory cytokines (33). The ability of 4-OI to activate NRF2 has been implicated in several of its anti-inflammatory and protective functions (25, 34–38).

Dimethyl fumarate (DMF) is a derivative of another Krebs cycle metabolite, fumarate, that is clinically approved for the treatment of multiple sclerosis (MS) (39). Like 4-OI, DMF is also a potent cysteine modifier (cysteine alkylation by fumarate is termed succination) (40), and DMF shares some of the same targets as 4-OI, such as GAPDH (27, 41), gasdermin D (28, 30, 42) and importantly KEAP1 (25, 43), meaning that DMF is also a potent NRF2 activator. Therefore these two metabolite derivates often have similar effects on biological pathways.

The effect of itaconate and its derivatives on PG production has not been studied to date. Here we show that 4-OI greatly reduces PG production in pro-inflammatory macrophages through transcriptional suppression of COX2. We demonstrate that PG production and COX2 transcription is unchanged by the deletion of IRG1, indicating a difference with endogenous itaconate. However, DMF replicates the decrease in COX2 expression and PG secretion observed with 4-OI. Finally we show that 4-OI and DMF reduce COX2 expression in an NRF2-independent manner. We have therefore uncovered a novel anti-inflammatory role of 4-OI and DMF in macrophages.

## Materials and Methods

### Reagents

4-Octyl itaconate was initially supplied by Professor Richard Hartley and results were later confirmed with commercially available 4-OI (Sigma Aldrich). Pam3CSK4, dimethyl fumarate, diethyl maleate, indomethacin and NS-398 (Sigma Aldrich) were also used. Antibodies used were anti-β-actin (Sigma Aldrich), anti-COX2 (Abcam), anti-phospho-cPLA2 (Ser505), anti-cPLA2, anti-NRF2, anti-KEAP1, anti-ATF4, anti-phospho-NF-kB p65 (Ser536), anti-NF-kB p65, anti-phospho-p38 MAPK (Thr180/Tyr182), anti-p38 MAPK, anti-phospho p44/42 MAPK (Erk1/2) (Thr202/Tyr 204) and anti-p44/42 MAPK (Erk1/2) (Cell Signaling). Anti-mouse IgG and anti-rabbit IgG secondary horseradish peroxidase-conjugated antibodies (Jackson Immunoresearch) were also used. A PGE_2_ ELISA kit was used (Enzo Life Sciences). The Silencer Select siRNAs against NRF2 (s70522), ATF4 (s62689) and Anxa1 (s69299), as well as the Silencer Select negative control, were used (ThermoFisher Scientific).

### Mice and BMDM generation

Bone marrow-derived macrophages (BMDMs) were isolated from C57BL/6J mice (Harlan UK). Legs from NRF2 knockout, KEAP1 knockdown and matched wild type mice were kindly provided by Professor Albeena Dinakova-Kostova (University of Dundee). The animals were housed under specific pathogen-free conditions in keeping with Irish and European Union regulations. All experiments carried out required prior ethical approval by Trinity College Dublin Animal Research Ethics Committee and Health Products Regulatory Authority. Mice were euthanised in a carbon dioxide chamber, after which cervical dislocation was used to confirm death. The ends of the tibia, femur and hip bones were cut and the bone marrow was flushed. The cells were then differentiated in Dulbecco’s modified Eagle’s medium (DMEM) containing 10% (vol/vol) foetal calf serum (FCS), 1% penicillin/streptomycin and 20% L929 supernatant for six days. After this time the macrophages were counted and replated for use in experiments.

### Human PBMC isolation

Thirty mL of whole blood was layered onto 20 mL Lymphoprep (Stemcell Technologies) in a 50 mL conical tube and centrifuged for 20 min at 2,000 rpm with no brake. The peripheral blood mononuclear cells (PBMCs) were then isolated from the middle layer and washed twice in PBS. PBMCs were cultured in RPMI supplemented with 10% (vol/vol) FCS and 1% penicillin/streptomycin for use in experiments.

### siRNA transfection

The media on the cells was replaced with 500 μL DMEM containing no FCS or penicillin/streptomycin. The required amount of Lipofectamine RNAiMAX transfection reagent (Thermo Fischer Scientific) and the siRNAs were diluted in DMEM containing no FCS or penicillin/streptomycin and pre-incubated together for 15 minutes. 500 μL of this mix was then added to each well so that the final dilution of Lipofectamine RNAiMAX was 5 μL/mL and the final concentration of siRNA was 50 nM.

### Western blotting

Cells were lysed in sample buffer [0.125 M Tris pH 6.8, 10% (vol/vol) glycerol, 0.02% SDS] and subsequently incubated at 95°C for a duration of five minutes. The samples were resolved on SDS-polyacrylamide gels, alongside the Spectra BR protein ladder (Thermofisher Scientific) so that proteins could be identified by molecular weight. The protein was then transferred to polyvinylidene fluoride membrane and membranes were blocked for one hour in 5% (w/v) dried milk in Tris-buffered saline/Tween (TBST). The blots were then incubated overnight at 4°C with the primary antibody. Following incubation for one hour with the secondary antibody, as well as three washes in TBST before and after secondary antibody, the blots were developed using chemiluminescent substrate (Thermofisher Scientific).

### Real time PCR

Cells were lysed and the RNA was extracted using the PureLink RNA minikit (Ambion). cDNA was subsequently prepared using the High Capacity cDNA Reverse Transcription kit (Applied Biosystems), according to manufacturer’s instructions. Real-time quantitative PCR (qPCR) was then performed with the resulting cDNA using a 7500 Fast Real-Time PCR System with PowerUp SYBR Green Master Mix (Applied Biosystems). All genes were normalised to *Rps18* expression for mouse or *RPS13* for human. The sequences of the primer pairs for murine genes that were used are as follows; *Rsp18*, 5’-GGA TGT GAA GGA TGG GAA GT-3’ (forward) and 5’-CCC TCT ATG GGC TCG AAT TT-3’ (reverse); *Ptgs2*, 5’CGG ACT GGA TTC TAT GGT GAA A-3’ (forward) and 5’CTT GAA GTG GGT CAG GAT GTA G-3’ (reverse); *Ptges*, 5’GGA AGA AGG CTT TTG CCA ACC-3’ (forward) and 5’-CGA AGC CGA GGA AGA GGA AA-3’ (reverse); *Nqo1*, 5’-GCT GCA GAC CTG GTG ATA TT-3’ (forward) and 5’-ACT CTC TCA AAC CAG CCT TT-3’ (reverse); *Il1b*, 5’-GGA AGC AGC CCT TCA TCT TT-3’ (forward) and 5’-TGG CAA CTG TTC CTG AAC TC-3’ (reverse); *Il6*, 5’-CCA CAG TCC TTC AGA GAG ATA CA-3’ (forward) and 5’-CCT TCT GTG ACT CCA GCT TAT C-3’ (reverse); *Tnf*, 5’-GCC TCT TCT CAT TCC TGC TT-3’ (forward) and 5’-TGG GAA CTT CTC ATC CCT TTG-3’ (reverse); *Il10*, 5’-AGG CGC TGT CAT CGA TTT-3’ (forward) and 5’-CAC CTT GGT CTT GGA GCT T-3’ (reverse); *Tgfb1*, 5’-CGA AGC GGA CTA CTA TGC TAA A-3’ (forward) and 5’-TCC CGA ATG TCT GAC GTA TTG-3’ (reverse); *Hmox1*, 5’-CCT CAC AGA TGG CGT CAC TT-3’ (forward) and 5’-GCT GAT CTG GGG TTT CCC TC-3’ (reverse); *Nos2*, 5’-TTC ACC CAG TTG TGC ATC GAC CTA-3’ (forward) and 5’-TCC ATG GTC ACC TCC AAC ACA AGA-3’ (reverse); *Ccl2*, 5’-GTT GGC TCA GCC AGA TGC A-3’ (forward) and 5’-AGC CTA CTC ATT GGG ATC ATC TTG-3’ (reverse); *Cd86*, 5’-TCT CCA CGG AAA CAG CAT CT-3’ (forward) and 5’-CTT ACG GAA GCA CCC ATG AT-3’ (reverse). The sequences of the primer pairs for human genes that were used are as follows; *RPS13*, 5’- TCA CCG TTT GGC TCG ATA TT-3’ (forward) and 5’-GGC AGA GGC TGT AGA TGA TT-3’ (reverse); *PTGS2* 5’-TGC GCC TTT TCA AGG ATG GA-3’ (forward) and 5’-CCC CAC AGC AAA CCG TAG AT-3’ (reverse); *IL1B*, 5’-AGC TGA TGG CCC TAA ACA GA-3’ (forward) and 5’-TGT CCA TGG CCA CAA CAA CTG A-3’ (reverse); *IL6*, 5’-TCT GGA TTC AAT GAG GAG ACT TG-3’ (forward) and 5’-CTC AAA TCT GTT CTG GAG GTA CT-3’ (reverse); *TNF*, 5’-CCA GGG ACC TCT CTC TAA TCA-3’ (forward) and 5’-TCA GCT TGA GGG TTT GCT AC-3’ (reverse); *IL10*, 5’-CTG TCA TCG ATT TCT TCC CTG T-3’ (forward) and 5’-TGC CTT TCT CTT GGA GCT TAT T-3’ (reverse); *TGFB1*, 5’-CTG CAC TAT TCC TTT GCC C-3’ (forward) and 5’-TCT TCT TCA CTA TCC CCC AC-3’ (reverse); *HMOX1*, 5’-CCC AGC CCT ACA CCC GCT AC-3’ (forward) and 5’-GGT GGC ACT GGC AAT GTT GG-3’ (reverse).

### ELISA

Cell supernatants were harvested and PG concentrations were quantified using an ELISA kit for PGE_2_ (Enzo Life Sciences), according to the manufacturer’s instructions. This item is sold as a PGE_2_-specific ELISA kit, however, our data indicated that other COX-derived oxylipins were also likely to be detected with this kit. Therefore we refer to results obtained using this ELISA kit as measuring PGs instead of PGE_2_.

### Oxylipin analysis

Immediately upon harvesting, cell supernatants were snap frozen in liquid nitrogen. Samples were spiked with 2.1-2.9ng of PGE_2_-d4, PGD_2_-d4, 20-HETE-d6 and TXB_2_-d4 standards (Cayman Chemical). Lipids were extracted by adding a 2.5 ml solvent mixture (1 M acetic acid/isopropanol/hexane; 2:20:30, v/v/v) to 1 ml supernatants in a glass extraction vial and vortexed for 30 sec. 2.5ml hexane was added to samples and after vortexing for 30 seconds, tubes were centrifuged (500 g for 5 min at 4 °C) to recover lipids in the upper hexane layer (aqueous phase), which was transferred to a clean tube. Aqueous samples were re-extracted as above by addition of 2.5 ml hexane, and upper layers were combined. Lipid extraction from the lower aqueous layer was then completed according to the Bligh and Dyer technique. Specifically, 3.75ml of a 2:1 ratio of methanol:chloroform was added followed by vortexing for 30 secs. Subsequent additions of 1.25ml chloroform and 1.25ml water were followed with a vortexing step for 30 seconds, and the lower layer was recovered following centrifugation as above and combined with the upper layers from the first stage of extraction. Solvent was dried under vacuum and lipid extract was reconstituted in 100μl HPLC grade methanol. Lipids were separated by liquid chromatography (LC) using a gradient of 30-100% B over 20 minutes (A: Water:Mob B 95:5 + 0.1% Acetic Acid, B: Acetonitrile: Methanol – 80:15 + 0.1% Acetic Acid) on an Eclipse Plus C18 Column (Agilent), and analysed on a Sciex QTRAP® 6500 LC-MS/MS system. Source conditions: TEM 475°C, IS -4500, GS1 60, GS2 60, CUR 35. Lipids were detected using MRM monitoring with the following parent to daughter ion transitions: PGE_2_ and PGD_2_ [M-H]- 351.2/271.1, 15-deoxy-PGJ2 [M-H]- 315.2/271.1, TXB_2_ [M-H]- 369.2/169.1. Deuterated internal standards were monitored using precursor to product ions transitions of: TXB_2_-d4 [M-H]- 373.2/173.1, PGE_2_-d4 and PGD_2_-d4 [M-H]-355.2/275.1, and 20-HETE-d6 [M-H]- 325.2/281.1. Chromatographic peaks were integrated using Multiquant 3.0.2 software (Sciex). The criteria for assigning a peak was signal:noise of at least 5:1 and with at least 7 points across a peak. The ratio of analyte peak areas to internal standard was taken and lipids quantified using a standard curve made up and run at the same time as the samples.

### Statistical Analysis

Statistical significance was established by the one-way or two-way ANOVA methods as indicated in the figure legends. Data are expressed as mean ± standard error of the mean. Significance was designated as follows: *p < 0.05, **p < 0.005, ***p < 0.0005, ****p < 0.0001. GraphPad Prism version 9 software was used for statistical analysis.

## Results

### 4-OI inhibits Pam3CSK4-induced PG and thromboxane secretion by macrophages

**Figure 1:**
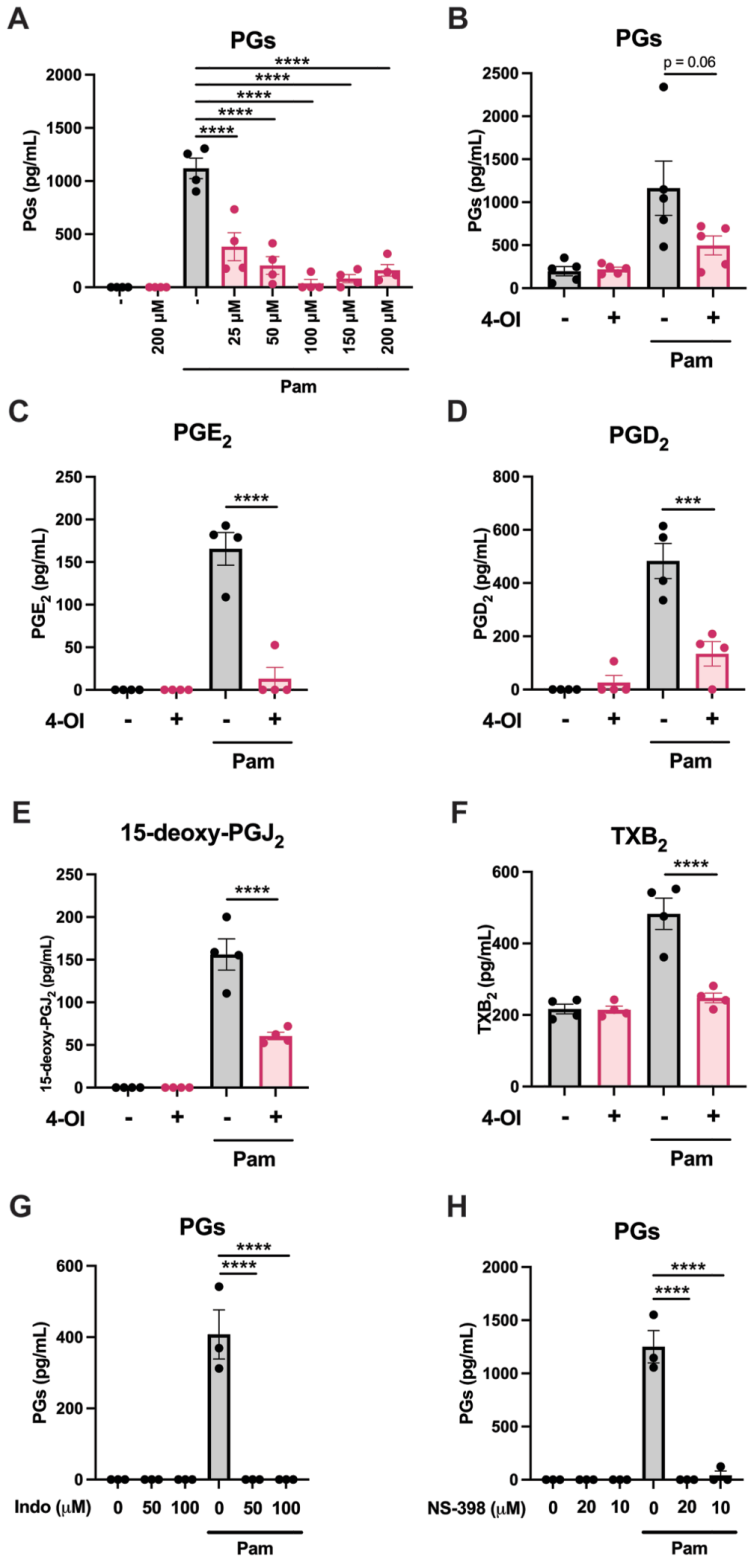
4-OI inhibits Pam3CSK4-induced prostaglandin production. (A) BMDMs were pretreated with various concentrations of 4-OI (25-200 μM) for two hours prior to stimulation with Pam3CSK4 (100 ng/mL) for 24 hours. The PG concentrations in the resulting supernatants were subsequently quantified by ELISA (n=4). (B) Human PBMCs were pretreated with 200 μM 4-OI for two hours prior to stimulation with Pam3CSK4 (1 μg/mL) for 24 hours. Supernatants were analysed for PG concentration by ELISA (n=5). (C-F) BMDMs were pretreated with 200 μM 4-OI for two hours prior to stimulation with Pam3CSK4 (100 ng/mL) for 24 hours. Supernatants were subsequently analysed by tandem mass spectrometry in order to determine (C) PGE_2_, (D) PGD_2_, (E) 15-deoxy-PGJ2 and (F) TXB_2_ concentrations (n=4). (G) BMDMs were pretreated with 50 μM or 100 μM indomethacin for one hour prior to stimulation with Pam3CSK4 (100 ng/mL) for 24 hours. The PG concentrations in the resulting supernatants were subsequently quantified by ELISA (n=3). (H) BMDMs were pretreated with 10 μM or 20 μM NS-398 for one hour prior to stimulation with Pam3CSK4 (100 ng/mL) for 24 hours. The PG concentrations in the resulting supernatants were subsequently quantified by ELISA (n=3). Data are mean ± S.E.M. *p < 0.05, **p < 0.005, ***p < 0.0005, ****p < 0.0001 by one-way ANOVA.

In order to investigate if 4-OI might play a role in modulation of prostaglandins, we treated murine BMDMs with 4-OI prior to stimulation with the TLR1/2 agonist Pam3CSK4 for 24 hours, which strongly induces PGs. We first used a PGE_2_ ELISA and observed a strong upregulation of cell supernatant PGs by Pam3CSK4, which was inhibited by 4-OI at concentrations as low as 25 μM (Figure 1A). 4-OI also reduced Pam3CSK4-induced PG secretion by human PBMCs (Figure 1B). To validate this result, we performed a lipidomic screen of oxylipins in BMDMs using quantitative tandem mass spectrometry (LC/MS/MS). Again, stimulation with Pam3CSK4 lead to a robust increase in PGE_2_ that was blocked by 4-OI pretreatment (Figure 1C), however the concentrations were notably lower than those measured by ELISA (Figure 1A). This is likely because of antibody-based measurements for lipid measurements displaying lower specificity and reporting on COX-derived oxylipins more broadly. From the LC/MS/MS screen we also observed that 4-OI potently inhibited Pam3CSK4-induced increases in PGD_2_ (Figure 1D), 15-deoxy-PGJ2 (Figure 1E) and thromboxane B_2_ (TXB_2_) (Figure 1F), in addition to PGE_2_. Therefore 4-OI was inhibiting all oxylipins downstream of COX activity. As confirmation that the ELISA was detecting COX-derived PGs in general, we found that the COX inhibitor indomethacin completely inhibited the Pam3CSK4-induced lipids from BMDMs detected by the ELISA (Figure 1G). In addition, we used the COX2-specific inhibitor NS-398, which also inhibited all Pam3CSK4-induced lipids detected by the ELISA (Figure 1H), indicating that PG secretion from BMDMs is almost entirely dependent on COX2.

### 4-OI suppresses Pam3CSK4-induced COX2 expression

**Figure 2:**
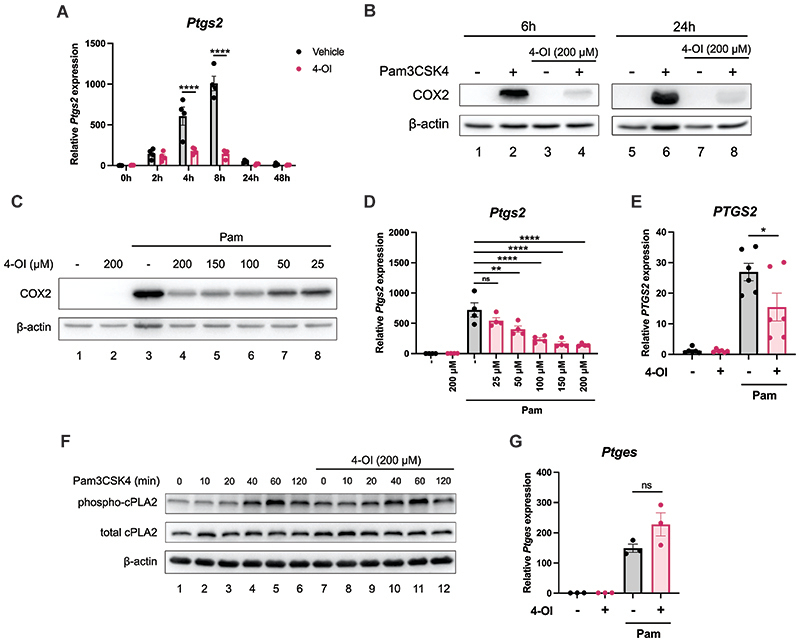
4-OI inhibits Pam3CSK4-induced COX2 expression. (A) BMDMs were pretreated with 200 μM 4-OI prior to stimulation with Pam3CSK4 (100 ng/mL) for 2, 4, 8, 24 or 48 hours. The cells were lysed, mRNA was extracted and *Ptgs2* expression was measured by qPCR (n=4) (B) BMDMs were pretreated with 200 μM 4-OI prior to stimulation with Pam3CSK4 (100 ng/mL) for 6 hours or 24 hours. COX2 expression was analysed by Western blotting (n=6). (C) BMDMs were pretreated with various concentrations of 4-OI (25-200 μM) prior to stimulation with Pam3CSK4 (100 ng/mL) for 24 hours. COX2 expression was analysed by Western blotting (n=4). (D) BMDMs were pretreated with various concentrations of 4-OI (25-200 μM) prior to stimulation with Pam3CSK4 (100 ng/mL) for six hours. After cell lysis, mRNA was extracted and *Ptgs2* expression was measured by qPCR (n=4). (E) PBMCs were pretreated with 200 μM 4-OI for two hours prior to stimulation with Pam3CSK4 (1 μg/mL) for six hours. The cells were then lysed, mRNA was extracted and *PTGS2* expression was measured by qPCR (n=6). (F) BMDMs were pretreated with 200 μM 4-OI prior to stimulation with Pam3CSK4 (100 ng/mL) for various timepoints (10-120 minutes). Phospho-cPLA2 and total cPLA2 expression was analysed by Western blotting (n=3). (G) BMDMs were pretreated with 200 μM 4-OI prior to stimulation with Pam3CSK4 (100 ng/mL) for four hours. After cell lysis, mRNA was extracted and *Ptges* expression was measured by qPCR (n=3). Data are mean ± S.E.M. *p < 0.05, **p < 0.005, ***p < 0.0005, ****p < 0.0001, ns = non-significant, by one-way ANOVA.

As we had observed that 4-OI suppressed all detected oxylipins downstream of COX activity, we next investigated if 4-OI had an effect on COX2 expression. COX2 is potently upregulated by TLR ligands such as Pam3CSK4, as seen by upregulation of *Ptgs2* transcript, the gene that encodes COX2, as well as strong boost in COX2 protein levels. 200 μM 4-OI potently blocked Pam3CSK4-induced *Ptgs2* levels at four hours and eight hours, the timepoints with the greatest induction of *Ptgs2* (Figure 2A). 4-OI also greatly decreased COX2 protein levels at both six hours and 24 hours (Figure 2B). Concentrations as low as 25 μM 4-OI reduced COX2 protein at 24 hours (Figure 2C), while concentrations 50 μM or higher decreased *Ptgs2* transcript at six hours (Figure 2D). 4-OI also reduced transcript levels of *PTGS2* in human PBMCs (Figure 2E). The reduction of COX2 expression with 4-OI is likely the reason that 4-OI inhibits PG secretion, as COX2 is often referred to as the rate-limiting enzyme for PG synthesis. 4-OI did not alter the phosphorylation levels of cPLA2, nor did it affect total cPLA2 (Figure 2F). 4-OI also had no significant effect on the mRNA levels of *Ptges* (Figure 2E), the gene that encodes prostaglandin E2 synthase (PGES), the enzyme that catalyses conversion of PGH_2_ to PGE_2_. Therefore, on the PGE_2_ biosynthetic pathway 4-OI seems to specifically suppress COX2 expression, which leads to a decrease in downstream PG production.

As has been previously reported (25, 27), we observed that 4-OI modulated the mRNA expression of several cytokine genes in BMDMs and PBMCs that were induced by Pam3CSK4 stimulation (Supplemental Figure 1A-E, J-N), such as yielding a reduction in *Il1b, Il6 and Il10* transcripts. 4-OI also reduced mRNA levels of *Nos2*, which encodes nitric oxide synthase (Supplemental Figure 1G), and the chemokine *Ccl2* (Supplemental Figure 1H). As expected, 4-OI increased transcript levels of the NRF2-dependent gene *Hmox1* in both BMDMs and PBMCs (Supplemental Figure 1F and O respectively).

### Endogenous itaconate does not affect COX2 expression or PG production

**Figure 3:**
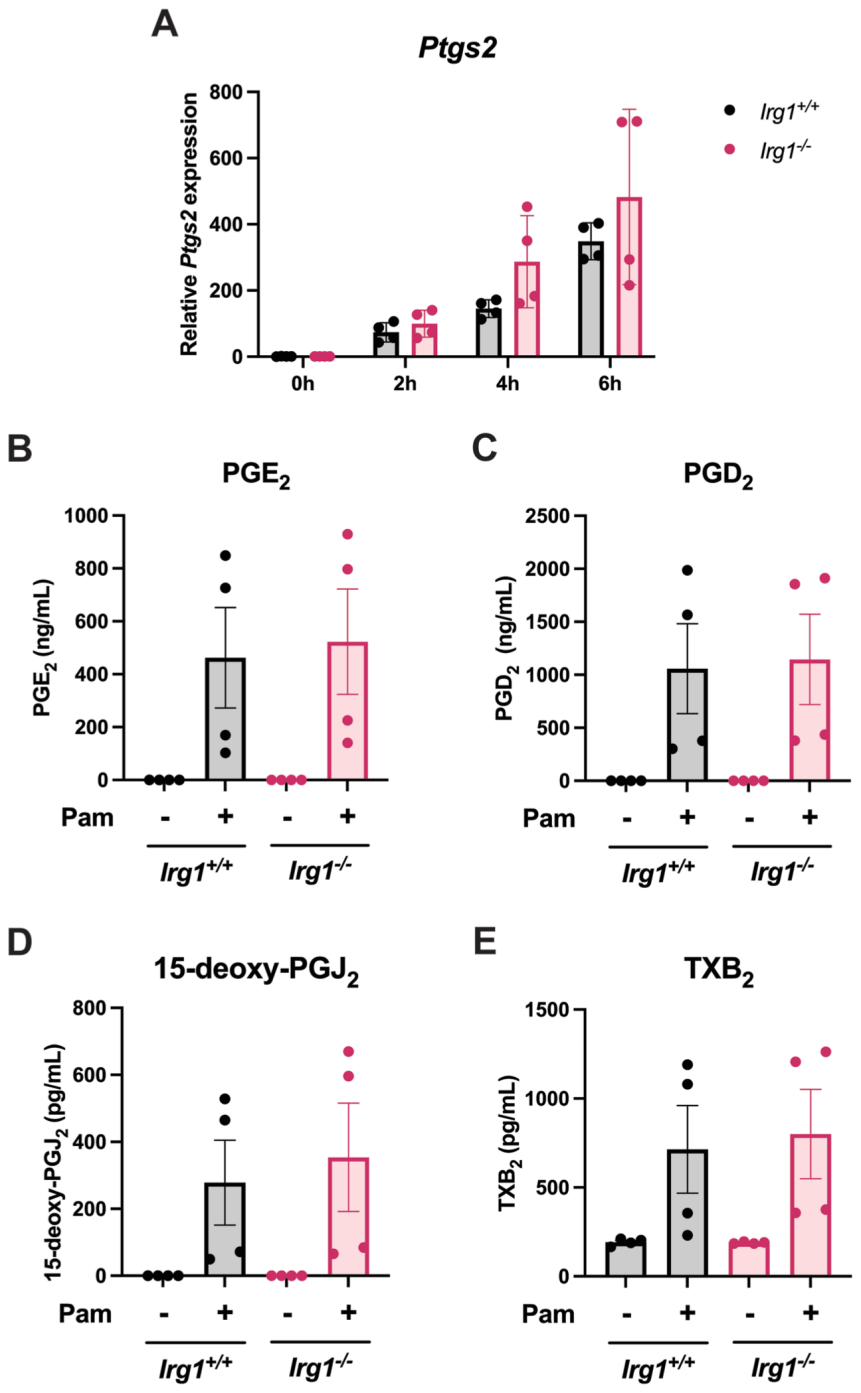
Endogenous itaconate does not affect COX2 expression or prostaglandin production. (A) BMDMs from *Irg1^+/+^* and *Irg1^-/-^* mice were stimulated with Pam3CSK4 (100 ng/mL) for two, four or six hours. The cells were lysed and mRNA extracted in order to quantify *Ptgs2* by qPCR (n=4). (B-E) BMDMs from *Irg1^+/+^* and *Irg1^-/-^* mice were stimulated with Pam3CSK4 (100 ng/mL) for 24 hours. The cell supernatants were then analysed by tandem mass spectrometry in order to determine (B) PGE_2_, (C) PGD_2_, (D) 15-deoxy-PGJ2 and (E) TXB_2_ concentrations (n=4). Data are mean ± S.E.M. *p < 0.05, **p < 0.005, ***p < 0.0005, ****p < 0.0001 by one-way ANOVA, or two-way ANOVA for (A).

We next tested if endogenous itaconate, as well as the derivatised 4-OI, would impact COX2 expression and PG synthesis. For this we used BMDMs lacking *Irg1*, the gene that encodes the enzyme responsible for itaconate synthesis. However, when *Ptgs2* transcript levels between *Irg1^+/+^* BMDMs and *Irg1^-/-^* BMDMs are compared, there is no difference in COX2 induction by Pam3CSK4 (Figure 3A). In addition, there were no changes in the COX-derived oxylipins PGE_2_ (Figure 3B), PGD_2_ (Figure 3C), 15-deoxy-PGJ2 (Figure 3D) and TXB_2_ (Figure 3E) when measured by tandem mass spectrometry. This shows that endogenous itaconate does not affect COX2 expression or PG production.

### DMF also reduces Pam3CSK4-induced COX2 expression and PG production

**Figure 4:**
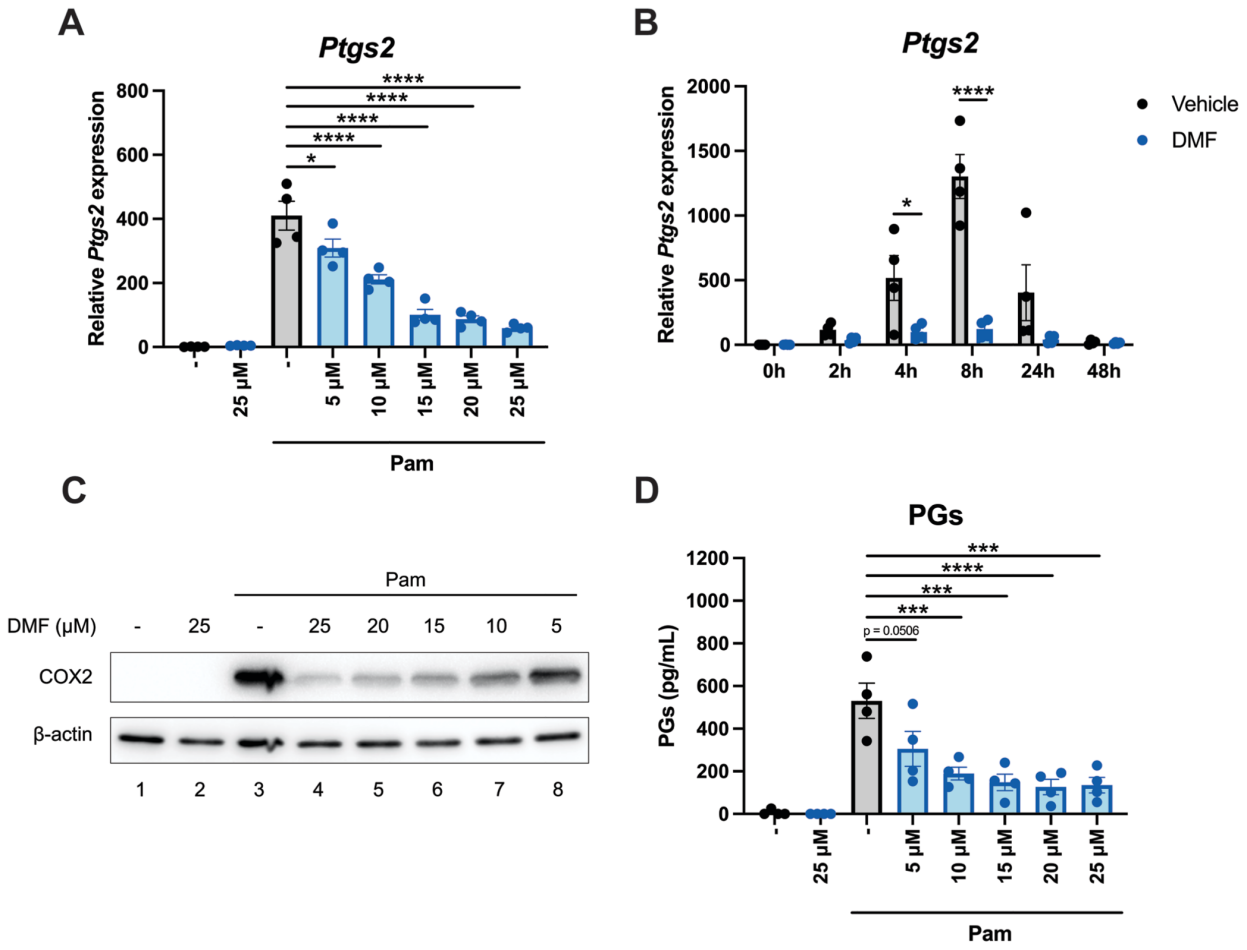
DMF decreases COX2 expression and prostaglandin production. (A) BMDMs were pretreated with various concentrations of DMF (5-25 μM) for two hours prior to stimulation with Pam3CSK4 (100 ng/mL) for four hours. After cell lysis, mRNA was extracted and *Ptgs2* levels were quantified by qPCR (n=4). (B) BMDMs were pretreated with 25 μM DMF for two hours prior to stimulation with Pam3CSK4 (100 ng/mL) for 2, 4, 8, 24 or 48 hours. After cell lysis, mRNA was extracted and *Ptgs2* levels were quantified by qPCR (n=4). (C) BMDMs were pretreated with various concentrations of DMF (5-25 μM) for two hours prior to stimulation with Pam3CSK4 (100 ng/mL) for 24 hours. COX2 expression was analysed by Western blotting (n=4). (D) BMDMs were pretreated with various concentrations of DMF (5-25 μM) for two hours prior to stimulation with Pam3CSK4 (100 ng/mL) for 24 hours. The PG concentrations in the resulting supernatants were subsequently quantified by ELISA (n=4). Data are mean ± S.E.M. *p < 0.05, **p < 0.005, ***p < 0.0005, ****p < 0.0001 by one-way ANOVA.

We next tested whether DMF might have similar effects on COX2 and PG production to 4-OI. Pretreatment of BMDMs with concentrations as low as 5 μM of DMF decreased levels of *Ptgs2* transcript (Figure 4A). DMF significantly downregulated *Ptsg2* mRNA levels at four hours and at eight hours (Figure 4B), similar to 4-OI. COX2 protein levels were also attenuated by DMF, using concentrations as low as 5 μM (Figure 4C). DMF also decreased Pam3CSK4-induced PG secretion by BMDMs (Figure 4D). Given that 4-OI and DMF are both potent cysteine modifiers and have very similar effects regarding COX2 expression and PG synthesis, it is likely that they are acting through a shared mechanism.

### The 4-OI- and DMF-induced suppression of COX2 and PGs is NRF2-independent

**Figure 5:**
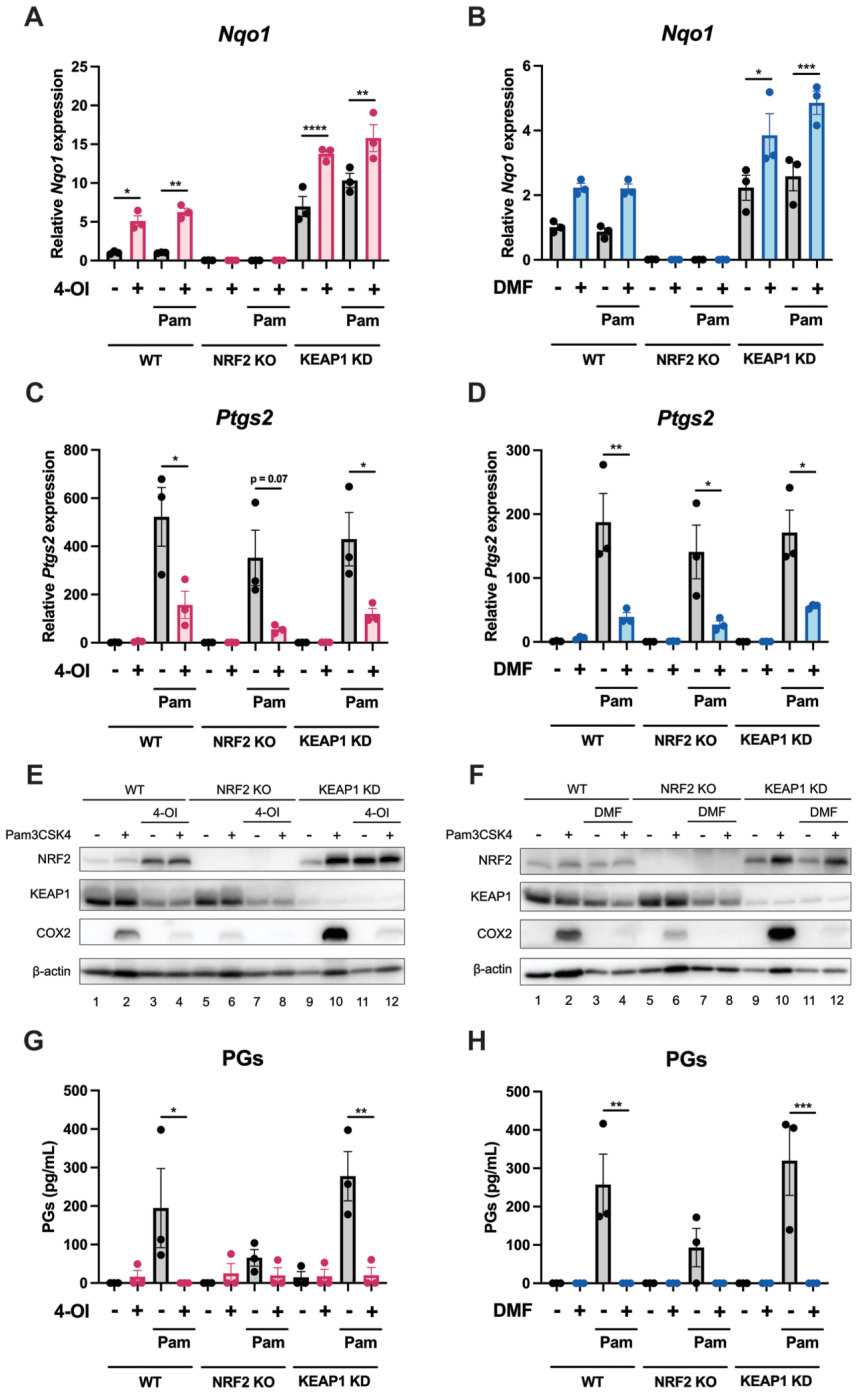
The capacity of 4-OI and DMF to reduce COX2 expression and prostaglandin production is not NRF2-dependent. (A-D) BMDMs from wild-type, NRF2 knockout and KEAP1 knockdown mice were pretreated with 200 μM 4-OI (A and C) or 25 μM DMF (B and D) for two hours prior to stimulation with Pam3CSK4 (100 ng/mL) for six hours. The cells were lysed, mRNA was extracted and *Nqo1* expression (A and B) and *Ptgs2* expression (C and D) were quantified by qPCR (n=3). (E-H) BMDMs from wild-type, NRF2 knockout and KEAP1 knockdown mice were pretreated with 200 μM 4-OI (E and G) or 25 μM DMF (F and H) for two hours prior to stimulation with Pam3CSK4 (100 ng/mL) for 24 hours. COX2 expression was analysed by Western blotting (E and F) (n=3). The supernatants were analysed for PG concentration by ELISA (G and H) (n=3). Data are mean ± S.E.M. *p < 0.05, **p < 0.005, ***p < 0.0005, ****p < 0.0001 by one-way ANOVA.

4-OI and DMF are both known to be potent NRF2 activators through their ability to modify crucial cysteine residues on KEAP1, a negative regulator of NRF2. We also tested if another NRF2 activating compound, diethyl maleate, DEM, (44) would also modulate COX2. Indeed pretreatment of BMDMs with DEM resulted in decreased COX2 protein levels (Supplemental Figure 2A) and transcription (Supplemental Figure 2B), in addition to suppression of PG secretion (Supplemental Figure 2C). This indicated that perhaps NRF2 was somehow involved in the modulation of COX2 and PGs. To test this, we used BMDMs isolated from wild-type, NRF2 knockout and KEAP1 knockdown mice. As expected, expression of the NRF2-dependent gene *Nqo1* was completely ablated in the NRF2 knockout BMDMs whereas the KEAP1 knockdown BMDMs displayed enhanced *Nqo1* transcription compared to wild type cells (Figure 5A and B, Supplemental Figure 2D). Both 4-OI and DMF maintained the capacity to suppress *Ptgs2* transcription in NRF2 knockout and KEAP1 knockdown BMDMs (Figure 5C and D respectively). 4-OI and DMF also reduced COX2 protein levels in NRF2 knockout and KEAP1 knockdown BMDMs (Figure 5E and F respectively). NRF2 knockdown using siRNA also did not abolish the capacity of 4-OI to reduce COX2 expression (Supplemental Figure 3A). Furthermore, 4-OI and DMF both blocked PG production in all genotypes (Figure 5G and H respectively). It is also worth noting that cells lacking NRF2 have lower levels of COX2 and PGs, whereas the KEAP1 knockdown cells, which exhibit augmented NRF2 activation, display elevated COX2 expression and PG production (Figure 5E-H). Interestingly, DEM, which is considered to be a well characterised NRF2 activator, could also still block *Ptgs2* transcription in NRF2 knockout and KEAP1 knockdown BMDMs (Supplemental Figure 2E), indicating that its ability to reduce COX2 is separate to its NRF2-activating function. We also observed that 4-OI had no effect on NF-kB p65 phosphorylation, p38 phosphorylation or ERK phosphorylation (Supplemental Figure 3B), all of which are signalling pathways that are known to modulate *ptgs2* transcription. We also investigated if ATF4 was involved in the observed effect, given that ATF4 was recently shown to bind directly to the *ptgs2* promoter and induce its transcription (45). However, 4-OI actually increased ATF4 expression and ATF4 silencing had no effect on the inhibition of COX2 by 4-OI (Supplemental Figure 3C). We also tested if the effect of 4-OI on COX2 might be dependent on annexin A1, which has been shown to be modified by itaconate and itaconate derivatives (25, 28, 29) and is known to function as a negative regulator of cPLA2, which catalyses the first step of PG biosynthesis. However, silencing annexin A1 did not alter the inhibition of PG production by 4-OI (Supplemental Figure 3D). These results indicate that the capacity of 4-OI and DMF to suppress COX2 expression and downstream PG production is independent of NRF2 activation and other known signals that regulate COX2.

## Discussion

The itaconate derivative 4-OI has recently garnered much attention as an immunomodulator. The findings of this study suggest a role for 4-OI in the inhibition of PG production in macrophages activated with the TLR1/2 ligand Pam3CSK4. We show that 4-OI potently reduces several COX-derived oxylipins and provide evidence that 4-OI suppresses COX2 transcription. We demonstrate that while endogenous itaconate derived from IRG1 activity does not affect COX2 levels, DMF attenuated COX2 expression and PG secretion in a similar manner to 4-OI. This implies that a cysteine modification may be involved but we also provide evidence that suggests that the effect of 4-OI and DMF on PG production is not via KEAP1 degradation and NRF2 activation. Further work is required in order to fully elucidate the mechanism by which 4-OI and DMF impair COX2 transcription.

Our knowledge of how Krebs cycle activity impacts inflammation has been rapidly expanding over the last number of years. Some Krebs cycle intermediates are known to exert proinflammatory actions, such as succinate which has been shown to stabilise HIF-1α, thereby upregulating IL-1β transcription (46). Succinate has also been reported to exacerbate certain inflammatory diseases, such as arthritis (47) and type 2 diabetes (48). Another Krebs cycle intermediate, citrate, has also been shown to alter the inflammatory response. The mitochondrial export, in addition to the breakdown of citrate, have been reported to be essential for nitric oxide and prostaglandin production (49, 50). Several anti-inflammatory roles have recently been described for both itaconate (25, 30, 51) and fumarate (41, 42) and here we report a novel function for derivatives of these two Krebs cycle metabolites in macrophages.

This work also highlights the importance of bearing in mind that metabolite derivatives do not always truly represent the action of the corresponding endogenous metabolites. In the case of 4-OI, the use of this derivative replicates the biological effects of endogenous itaconate in a number of cases such as the inactivation of the NLRP3 inflammasome (24) and impairment of glycolysis (27). However there are also incidences where 4-OI and endogenous itaconate differ in their effects, such as type I interferon (IFN) production, which is boosted by endogenous itaconate (52) but inhibited by 4-OI (25). While itaconate has also been shown to modify proteins in the same manner as 4-OI, the targets are not always the same. Our previous study (25) found that, while there were some overlapping proteins modified by both 4-OI and endogenous itaconate, many targets were mutually exclusive. This could potentially be due to differences in electrophilicity between itaconate and 4-OI.

Although DMF is known to be a potent NRF2 activator (43, 53), NRF2-independent anti-inflammatory functions of DMF have begun to emerge, such as the impairment of glycolysis through GAPDH succination (41) and inhibition of pyroptosis via gasdermin D (42). It is also true for 4-OI that a number of its currently known anti-inflammatory effects are NRF2-dependent (25, 34, 35), although others are not (24, 27, 30, 51). Interestingly, DEM, which is predominantly used as an experimental tool to pharmacologically activate NRF2, also supressed COX2 expression in an NRF2-independent manner. As DEM has been shown to modify cysteines on KEAP1 in a similar way to 4-OI and DMF (44), perhaps DEM also possesses the capacity to modify other reactive cysteines in the cell with further reaching implications. An observation that adds complexity to this system is that 15-deoxy-Δ^12,14^-PGJ2, which is downstream of COX activity and therefore would be reduced by 4-OI, has been shown to activate NRF2 (54). Nonetheless, our data reveals another NRF2-independent anti-inflammatory role for DMF, 4-OI and even DEM.

There is also the possibility that the effect of 4-OI on COX2 and prostaglandins will contribute to the attenuation of other inflammatory markers. For example, macrophages express EP receptors (55) and therefore PGE_2_ can signal in an autocrine manner. We have previously shown that endogenous PGE_2_ acting via the EP2 receptor is required for induction of pro-IL-1β (56). Hence the inhibition of PGE_2_ secretion from the macrophage and subsequent binding to EP2 could potentially contribute to the 4-OI-induced decrease in pro-IL-1β that has been reported (25). PGE_2_ has also been shown to augment IL-6 production in macrophages (57, 58), another cytokine that is blocked by 4-OI treatment (25). Therefore inhibition of PGE_2_ by 4-OI could potentially be a factor in the downregulation of IL-6. Treatment of macrophages with PGE_2_ was shown to boost production of IL-10 in macrophages (59), an anti-inflammatory cytokine that is also downregulated by 4-OI. It is also conceivable that a reduction in PGs secreted by macrophages could contribute to the anti-inflammatory effects of 4-OI during *in vivo* models (24, 25, 27). Conversely, the capacity of 4-OI to attenuate the production of other proinflammatory mediators could potentially affect the induction of COX2. For example, IL-1β and nitric oxide, both of which are inhibited by 4-OI (25), have been reported to contribute to COX2 induction (60, 61).

Our results therefore identify 4-OI and DMF as inhibitors of prostaglandin synthesis in macrophages. This work further highlights the potential of 4-OI as a therapeutic anti-inflammatory treatment and supplements the current knowledge of the anti-inflammatory effects of DMF.

## Supplementary Material

Supplemental Figure S1-3

## Key Points

- 4-OI reduces COX2 expression and inhibits prostaglandin production in macrophages
- DMF also attenuates COX2 expression in macrophages and decreases prostaglandins
- The 4-OI- and DMF-induced decrease in COX2 and prostaglandins is NRF2-independent
