# Supplementary material for: 4-Octyl-itaconate and dimethyl fumarate inhibit COX2 expression and prostaglandin production in macrophages
Source: J Immunol. Author manuscript; Available in PMC 2022 Aug 8. (PMC7613254; doi:10.4049/jimmunol.2100488)
Supplement: Supplemental Figure S1-3 [file EMS150775-supplement-Supplemental_Figure_S1_3.pdf]

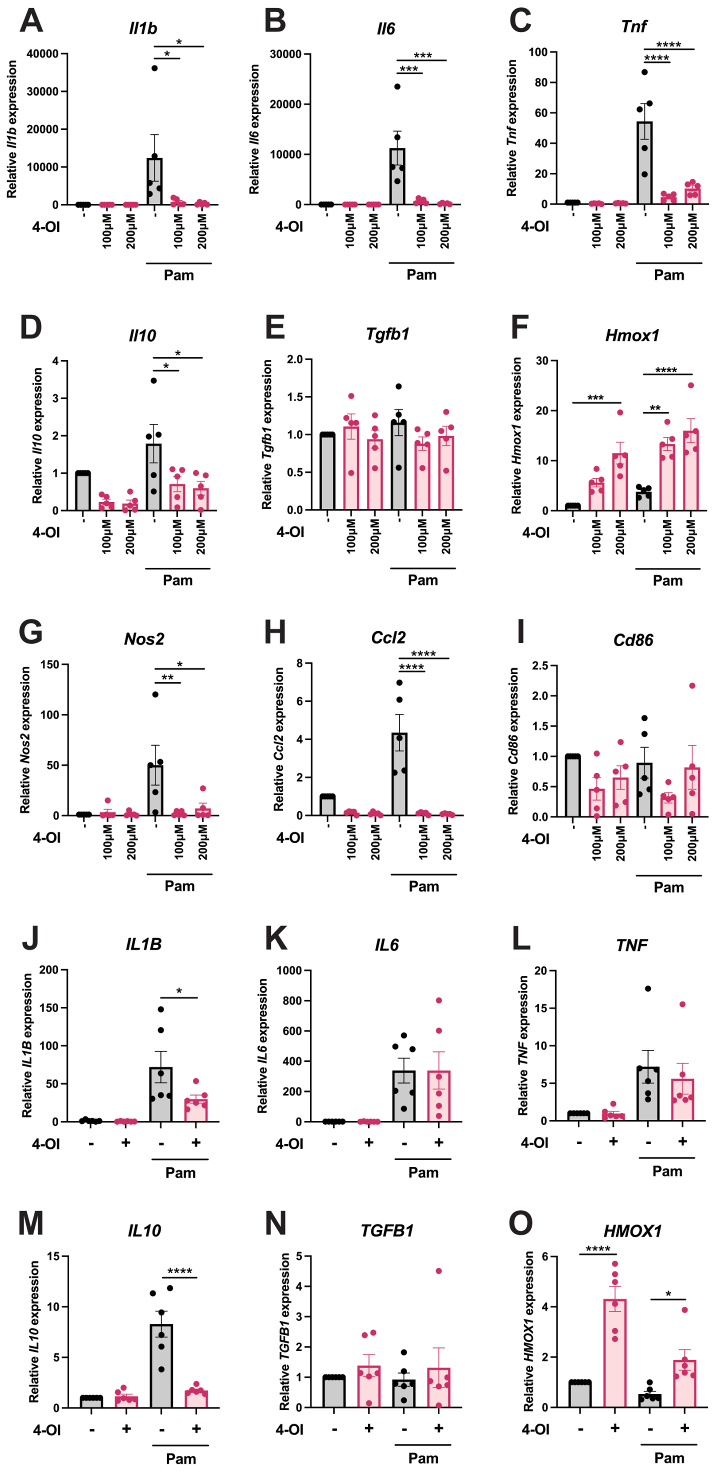

Supplemental Figure 1. 4-OI alters proinflammatory gene expression. (A-I) BMDMs were pretreated with DMSO or 4-OI (100 μM or 200 μM) for two hours prior to stimulation with Pam3CSK4 (100 ng/mL) for six hours. (A) *Il1b,* (B) *Il6,* (C) *Tnf,* (D) *Il10,* (E) *Tgfb1,* (F) *Hmox1,* (G) *Nos2,* (H) *Ccl2* and (I) *Cd86* expression were quantified by qPCR (n=5). (J-O) Human PBMCs were pretreated with DMSO or 200 μM 4-OI for two hours prior to stimulation with Pam3CSK4 (1 μg/mL) for six hours. (J) *Il1b,* (K) *Il6,* (L) *Tnf,* (M) *Il10,* (N) *Tgfb1* and (O) *Hmox1* expression were quantified by qPCR (n=6).

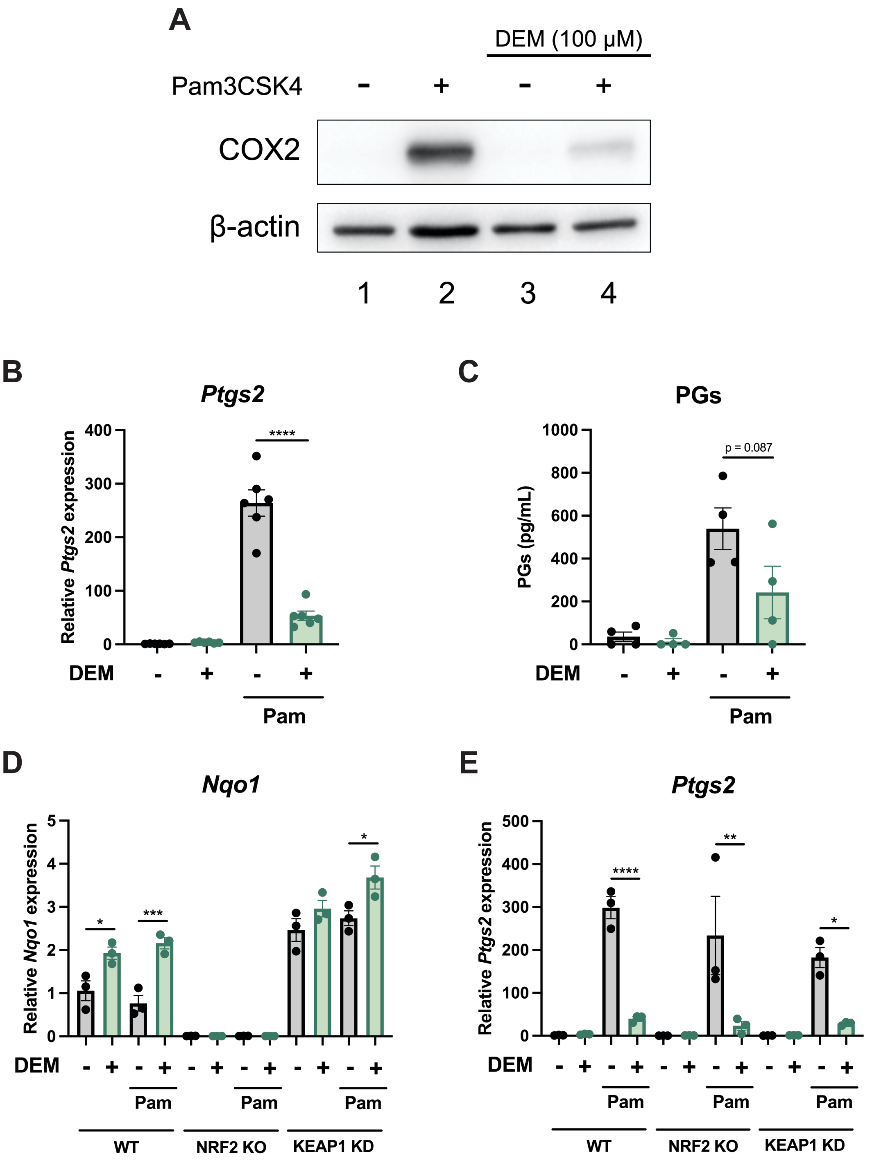

Supplemental Figure 2. DEM reduces COX2 expression and prostaglandin production in an NRF2-independent manner. (A) BMDMs were pretreated with 100 µM DEM for two hours prior to stimulation with Pam3CSK4 (100 ng/mL) for 24 hours. COX2 expression was analysed by Western blotting (n=4). (B) BMDMs were pretreated with 100 µM DEM for two hours prior to stimulation with Pam3CSK4 (100 ng/mL) for four hours. After cell lysis, mRNA was extracted and *Ptgs2* levels were quantified by qPCR (n=6). (C) BMDMs were pretreated with 100 µM DEM for two hours prior to stimulation with Pam3CSK4 (100 ng/mL) for 24 hours. The PG concentrations in the resulting supernatants were subsequently quantified by ELISA (n=4). (D and E) BMDMs from wild-type, NRF2 knockout and KEAP1 knockdown mice were pretreated with 100 µM DEM for two hours prior to stimulation with Pam3CSK4 (100 ng/mL) for six hours. The cells were lysed, mRNA was extracted and *Nqo1* expression (D) and *Ptgs2* expression (E) were quantified by qPCR (n=3). Data are mean ± S.E.M. *$p < 0.05$, **$p < 0.005$, ***$p < 0.0005$, ****$p < 0.0001$ by one-way ANOVA.

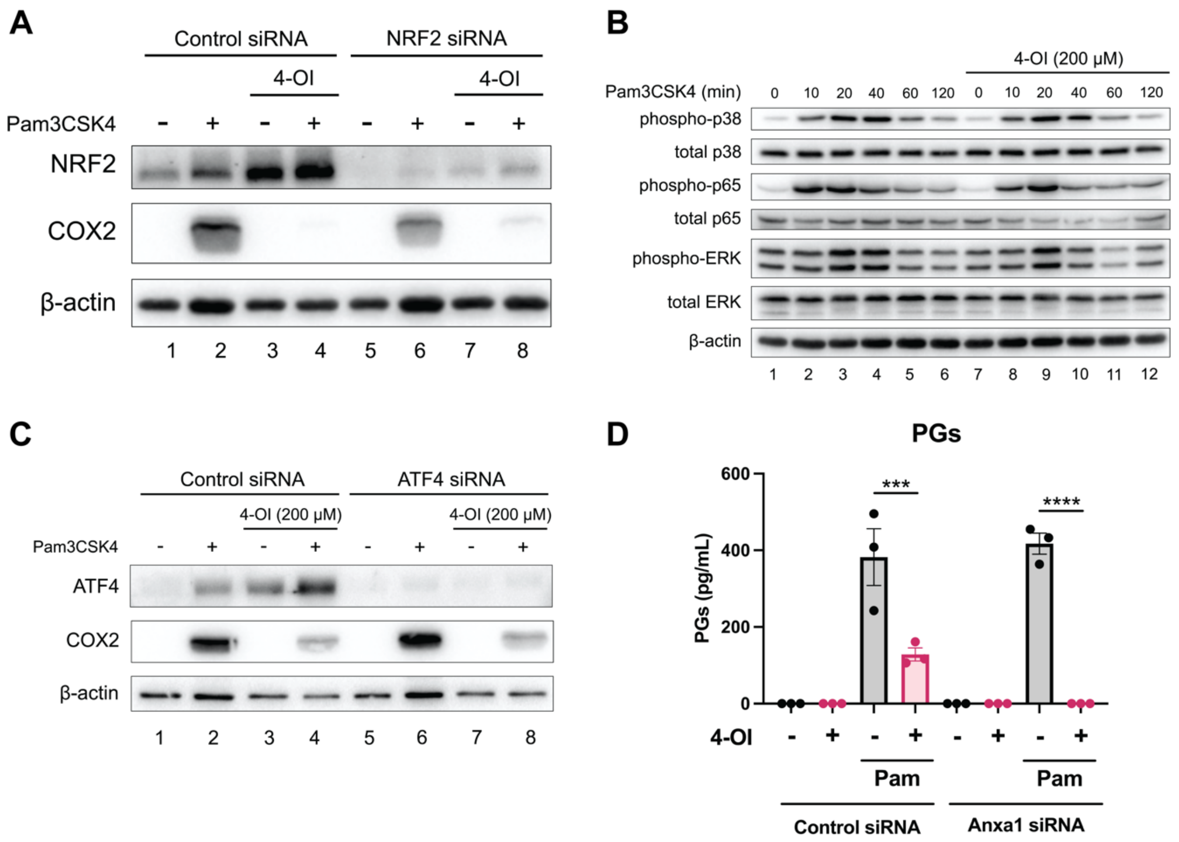

Supplemental Figure 3. The capacity of 4-OI to inhibit COX2 expression and prostaglandin production is not via NRF2, NF-kB, p38, ERK, ATF4 or annexin A1. (A) BMDMs were transfected with 50 nM control siRNA or NRF2 siRNA for 24 hours. The cells then were treated with 200 μM 4-OI prior to stimulation with Pam3CSK4 (100 ng/mL) for 24 hours. NRF2 and COX2 expression were analysed by Western blotting (n=5). (B) BMDMs were pretreated with 200 μM 4-OI prior to stimulation with Pam3CSK4 (100 ng/mL) for 10, 20, 40, 60 or 120 minutes. Phospho and total p38, p65 and ERK levels were measured by Western blotting (n=3). (C) BMDMs were transfected with 50 nM control siRNA or ATF4 siRNA for 48 hours. The cells then were treated with 200 μM 4-OI prior to stimulation with Pam3CSK4 (100 ng/mL) for 24 hours. ATF4 and COX2 expression were analysed by Western blotting (n=3). (D) BMDMs were transfected with 50 nM control siRNA or anxa1 siRNA for 96 hours. The cells then were treated with 200 μM 4-OI prior to stimulation with Pam3CSK4 (100 ng/mL) for 24 hours. The PG concentrations in the resulting supernatants were subsequently quantified by ELISA (n=3). Data are mean ± S.E.M. $^{*}p < 0.05$, $^{**}p < 0.005$, $^{***}p < 0.0005$, $^{****}p < 0.0001$ by one-way ANOVA.
